# Haplotype-resolved chromosome-level genome assemblies of nineteen apple (*Malus domestica* Borkh.) cultivars

**DOI:** 10.1038/s41597-026-06583-y

**Published:** 2026-01-24

**Authors:** Sophie Watts, Steven Yates, Stijn Vanderzande, Cecilia Hong Deng, Francesca Zuffa, Yutang Chen, Graham Dow, Bruno Studer, Giovanni Antonio Lodovico Broggini

**Affiliations:** 1 Molecular Plant Breeding, Institute of Agricultural Sciences, ETH Zurich, Universitaetstrasse 2, 8092 Zurich, Switzerland; 2 Plant Breeding, Wageningen University and Research, Wageningen, The Netherlands; 3 Mount Albert Research Centre, The New Zealand Institute for Plant and Food Research Limited, Auckland, New Zealand; 4 Crop Science and Production Systems, NIAB, Cambridge, UK

**Keywords:** Plant genetics, Natural variation in plants, Genome, Plant breeding

## Abstract

Apple (*Malus domestica* Borkh.) is a major fruit crop with a rich genetic history shaped by whole-genome duplication, domestication, and selective breeding. Discovering apple genetic diversity through genome sequencing provides new opportunities to improve disease resistance, environmental adaptation, and fruit quality. Here, we present 19 haplotype-resolved genome assemblies of apple, sequenced using PacBio HiFi reads with approximately 30 × coverage. Each haplome assembly has a mean length of 675.3 Mb and contains on average 47,445 annotated protein-coding genes. These haplome assemblies have a high completeness, with mean complete BUSCO scores of 98.8%. We identified 578 previously uncharacterized orthogroups shared across all 38 haplomes, indicating that these assemblies capture novel genetic diversity. Many of the assemblies are also highly contiguous, with on average three to four phase switches per chromosome. These data will accelerate genome-wide association studies, helping researchers to find and use genetic diversity for the improvement of key traits. Additionally, these data can offer insights into evolutionary history, domestication, and genetic diversity, supporting apple breeding and the broader *Rosaceae* research community.

## Background & Summary

Apple (*Malus domestica* Borkh.) is the third most valuable fruit crop grown globally^1^. The development of genomic resources, such as high-quality genome assemblies, can enable the dissection of key traits and lay the foundation for genomics-assisted breeding. Initial attempts at assembling apple genomes were, for a long-time, limited by the high degree of heterozygosity present in apple genotypes. The first whole genome sequence (WGS) for apple was reported for the cultivar ‘Golden Delicious’ and was generated using Sanger and 454 pyrosequencing technology^2^. An additional genome assembly of the cultivar ‘Golden Delicious’ was later published, using over 100-fold coverage Illumina short read combined with 29-fold coverage PacBio long read sequencing^3^. These assemblies were highly fragmented in part due to sequencing limitations and apple genome heterozygosity, as evidenced by a maximum N50 size of 111,619 bp^3^.

To bypass the technical challenges in assembling heterozygous genomes, a homozygous doubled haploid genotype of the apple cultivar ‘Golden Delicious’ was sequenced and assembled using a combination of Illumina short read, PacBio long read, and Bionano optical genome mapping technologies^4^. The resulting GDDH13v1.1 assembly had a genome contiguity (N50) that was an order of magnitude greater (5.5 Mb) than the former ‘Golden Delicious’ assembly (0.11 Mb) and served as the reference genome for apple. Consequently, researchers continued to exploit haploid accessions for subsequent genome assembly. In 2019, another homozygous line was sequenced, an anther-derived trihaploid ‘Hanfu’ (HFTH1) line, using Illumina short read, PacBio long read, Bionano optical mapping data, and Hi-C data for assembly^5^. In 2022, a homozygous tetra-haploid ‘Royal Gala’ plant was sequenced using Illumina short read, PacBio long read, and Hi-C library^6^. However, haploid assemblies of homozygous genotypes derived from anther culture only capture one of the two haplotypes from their heterozygous donors. With the rapid advances in long read sequencing technologies and assembly methods^7^, it is feasible to sequence heterozygous apple genotypes and generate haplotype–resolved assemblies containing both haplotypes. Haplotype-resolved genome assemblies distinguish both parental haplotypes, providing an improved representation of diploid species genomes, such as apple^8^.

The first attempt at a haplotype-resolved genome assembly was for the cultivar ‘Gala Galaxy’ using PacBio long reads for assembly, Illumina short reads for polishing, together with Bionano optical mapping for scaffolding^9^. Using the same methodology, along with a 10x Genomics library, the genomes of ‘Gala’ and two wild progenitors, *Malus sieversii* Ldb.and *Malus sylvestris* Mill., were assembled and phased^10^. This was followed by additional phased genome assemblies of the apple cultivars ‘Honeycrisp’, ‘Antonovka’, ‘Red Fuji’, and ‘WA 38'^11–14^. Recently, a haplotype-resolved assembly of the dwarfing apple interstock hybrid ‘SH6’ (*Malus honanensis* Rehder × *Malus domestica* Borkh.) and a telomere-to-telomere phased genome of assembly of ‘Golden Delicious’ were also published^15,16^. Furthermore, near-gapless haplotype-resolved assemblies for the dwarf rootstock ‘M9’, semi-rigorous rootstock ‘MM106’ and popular cultivar ‘Fuji’ were released^17^. These developments show that haplotype-resolved genome assemblies are a valuable resource for accurately characterizing highly heterozygous, diploid species, such as apple.

**Fig. 1:**
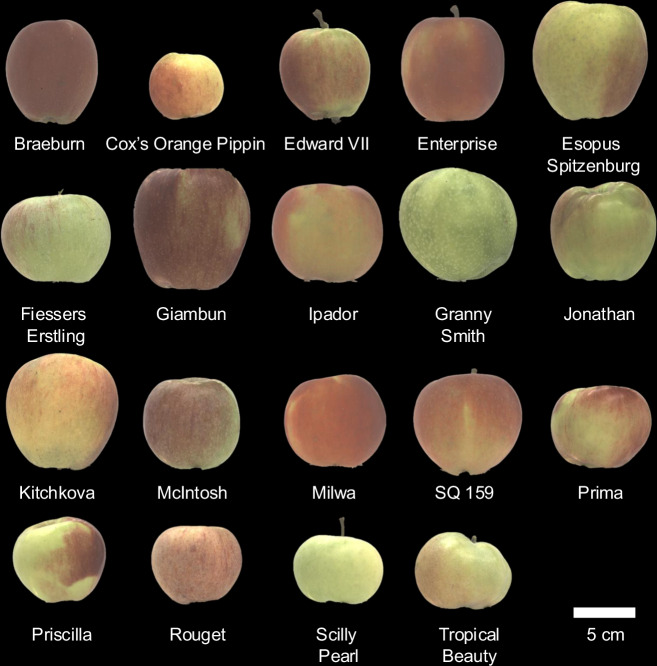
Photographs of apples from the cultivars that were sequenced in this study.

In this study, we sequenced 19 apple accessions (Fig. 1) using Pacific Bioscience’s high-fidelity sequencing technology. This resulted in 19 chromosome-level, haplotype-resolved genome assemblies, corresponding to 38 haplotype assemblies (haplomes). The haplome sizes ranged between 601.8 Mb to 767.1 Mb, with a mean of 675.3 Mb. The haplomes were highly complete with total complete Benchmarking Universal Single-Copy Orthologs (BUSCO) scores ranging between 93.1% to 99.3% ($\bar{x}$ = 98.8%). When evaluated with pedigree-phased high-quality single nucleotide polymorphism (SNP) array data, on average three to four phase switches were found per pseudo-chromosome, suggesting highly contiguous phasing. Protein-coding genes were annotated, and the number of gene models per haplome ranged between 43,278 to 49,666 ($\bar{x}$ = 47,445). Orthologous clustering of these proteins, with proteins from reference genome assemblies from GDDH13 v1.1, HFTH1 v1.0 and Honeycrisp v1.1.a1 (n = 1,988,899), resulted in defining 60,012 orthogroups^4,5,11^. Among the identified orthogroups, 13,985 were found to be shared across all haplotype-resolved chromosome-level genomes. These publicly available genomes are a resource for advancing genomic studies in apple with broader applications across Rosaceae species.

## Methods

### Samples collection, library construction and sequencing

**Fig. 2:**
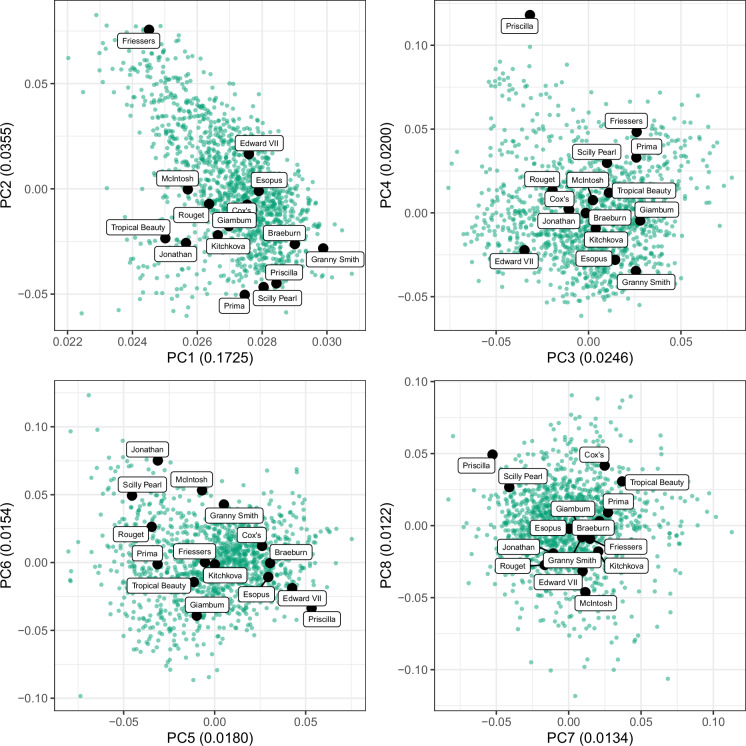
Principal component analysis (PCA) of REFPOP accessions based on 480 K SNP chip data. The original 480 K SNP chip dataset was converted to numeric format (0–1), with duplicated markers removed prior to analysis. PCA was performed using the ‘prcomp’ function in R. Green points represent all REFPOP accessions, while those labeled in black correspond to the accessions selected for WGS. The *x*- and *y*-axes represent principal components (PCs), with the variance explained indicated in parentheses. The four panels display the first eight PCs, which together account for 31% of the total variance.

**Fig. 3:**
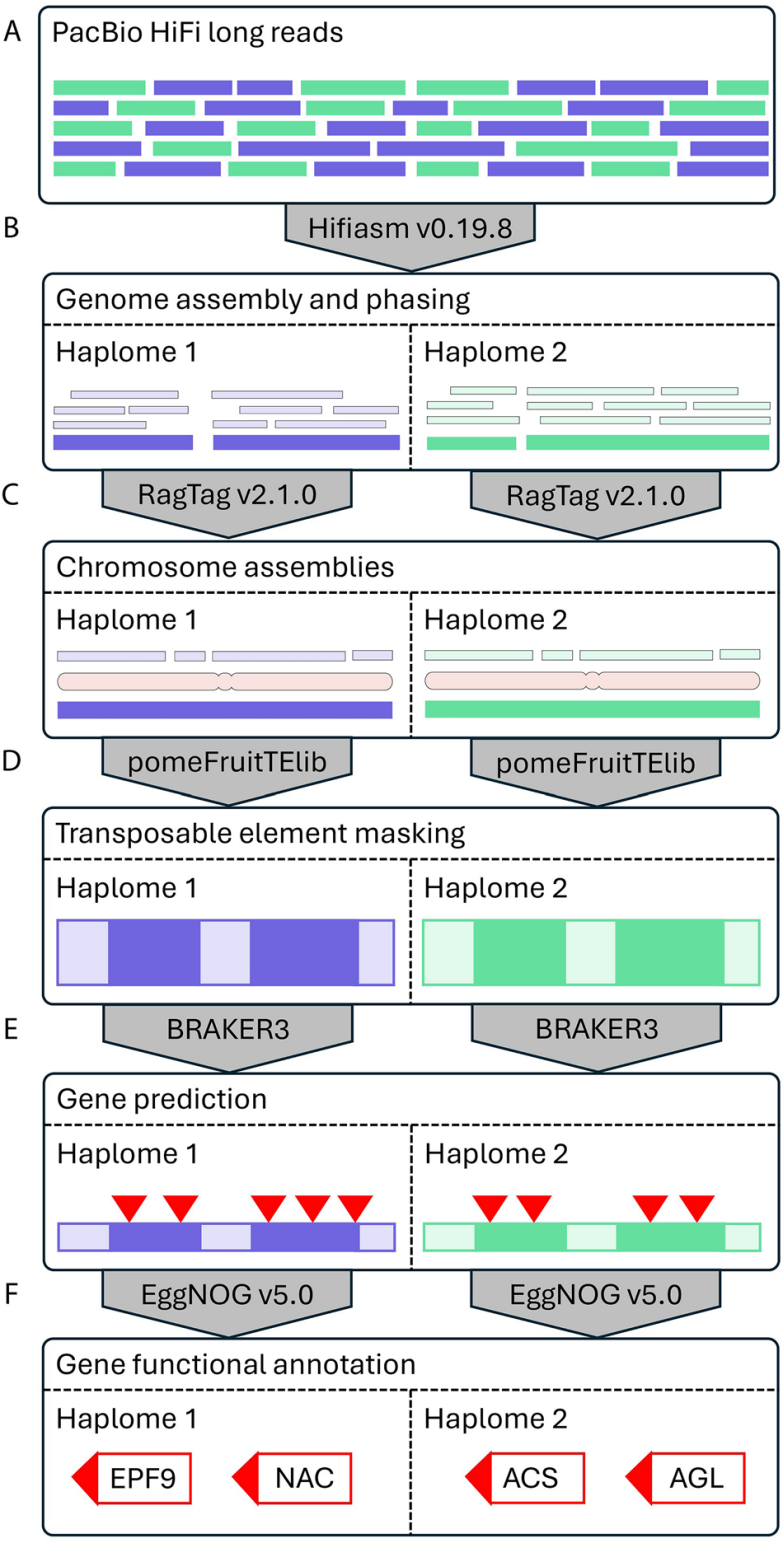
Bioinformatic workflow of the sequencing and assembly pipeline.

Nineteen apple accessions were selected for whole-genome sequencing (WGS), of which fourteen were drawn from the REFPOP^18^. Photographs of the apples collected from each accession are shown in Fig. 1. These accessions were chosen based on the following criteria: (i) phenotypic extremes: to represent the range of stomatal density, with accessions selected for high and low stomatal density as described by Zuffa *et al*.^19^ (ii) genetic diversity: to capture a broad spectrum of genetic diversity within the REFPOP, as illustrated by the inclusion of cultivars such as ‘Giambun’, ‘Prima’, ‘Rouget’, and ‘Tropical Beauty’ (Fig. 2) (iii) historical and commercial importance: to include cultivars of historical or commercial relevance, such as the cultivars ‘Granny Smith’ and ‘McIntosh’. The remaining five accessions were selected by breeders at Agroscope based on additional breeding and selection priorities. For WGS, approximately two grams of fresh growing leaves were harvested from the same tree of each accession in 2023. The sampled fresh leaves were flash frozen in liquid nitrogen, within one hour of collection and stored at −80 °C. The DNA extraction, library construction, and WGS were done by the Arizona Genomics Institute, USA, aiming for thirty-fold coverage per genome with the Revio system (Pacific Biosciences, Menlo Park, California, USA) to generate HiFi long reads. A workflow of the bioinformatic pipeline is shown in Fig. 3.

### Genome assembly

Raw data was converted from ‘bam’ to ‘fastq’ format using Samtools v1.19.2^20^. These data were then assembled using Hifiasm v0.19.8 and the resulting ‘asm’ haplome assemblies were extracted in ‘fasta’ format using Awk^21^. These assemblies were then organized into chromosomes based on the HFTH1 v1.0 reference genome using RagTag v2.1.0^5,22^, which incorporated Minimap2 v2.26 for alignments with the long assembly to reference mapping preset “-x asm5” and “-f 0.02”^23^.

Mitochondrial contamination errors were detected after uploading the haplome assemblies to NCBI (https://www.ncbi.nlm.nih.gov/home/genomes/) for quality assessment. The mitochondrial contaminants were removed from the genome using BEDTools v2.27.1^24^. The remaining sequences were extracted using SeqKit v2.4.0^25^, and concatenated together using Biopython v1.84^26^.

### Genome annotation

To annotate transposable elements (TEs) in the haplome assemblies, a pan-pome fruit transposable element library (pomeFruitTElib) was constructed based on six *Malus spp*. (GDDH13 v1.1^4^, HFTH1 v1.0^5^, Gala diploid Genome v1.0^10^, Royal Gala v1.0, *M. sieversii* v2, and *M. sylvestris* v2^6^), six *Pyrus spp*. (*P. communis* Bartlett DH v2^27^, *P. bretschneideri* v1.1^28^, *P. pyrifolia* Nijisseiki r.1.0.pmol^29^, *P. pyrifolia* Cuiguan v1.1^30^, *P. betuleafolia* v1.0^31^, and *P. ussuriensis* × *P. communis*, Zhongai v1.0^32^) and one *Gillenia* genome assembly^33^. The haplome assemblies were processed in parallel using the ‘genePal’ pipeline developed at The New Zealand Institute for Plant and Food Research Limited (https://github.com/Plant-Food-Research-Open/genepal), which includes repeat masking with pomeFruitTElib, *ab inito* gene prediction with BRAKER3^34^, liftoff gene models from ‘Viridiplantae’ in OrthoDB^35^ and published *Malus* assemblies (Honeycrisp v1.1.a1^11^, HFTH1 v1.0^5^, GDDH13 v1.1^4^), followed by functional annotation with EggNOG-mapper (https://github.com/eggnogdb/eggnog-mapper) on EggNOG v5.0 database^36^.

### Genome completeness

To assess the comprehensiveness of the assemblies, we used the BUSCO v5.1.2 tool, leveraging orthologous genes from the ‘embryophyta_odb10’ data^37^.

### Orthologous clustering

Orthologous relationships for genes were calculated using OrthoFinder v2.5.4^38,39^. For genes predicted in each haplome, the translated amino acid sequence of the primary transcript was selected as the representative protein. In addition, protein sequences from GDDH13 v1.1^4^, HFTH1 v1.0^5^, and Honeycrisp v1.1.a1^11^ were included in the orthologous analysis. An all-vs-all multiple sequence alignments of proteins from these genomes were performed with Mafft v7.307^40–42^. The OrthoFinder result was plotted using R v4.3.3^43^.

### Genome phasing quality check

The phasing quality of each haplome assembly was evaluated using the R-script developed by Vanderzande *et al*.^44^. Briefly, this reference SNP array data was obtained from the 20K SNP array^45^ and through other studies^46–48^ and was error-cleaned and phased according to Vanderzande *et al*.^49^. First, each haplome was compared to the iGL genetic map^46^, which is based on SNP array data^45^. Probe sequences for each SNP in this iGL map were aligned to each haplome using BLAST^50^ and the SNPs’ likely positions were determined according to Vanderzande *et al*.^44^. Then, per 2 cM interval of the iGL map, the proportion of SNPs not having a unique location in the haplome and the proportion of SNPs having a location inconsistent with the genetic map were recorded to indicate problematic regions where a haplome may have issues. Second, uniquely aligned SNPs that showed consistency with the iGL map were extracted from each haplome assembly and compared to the reference SNP array data. The proportion of genotypic inconsistencies between the SNP array data and alleles extracted from the assembly was determined to ensure the correct individual was sequenced. Furthermore, the phasing of the assembly was evaluated by comparing the SNP alleles from each haplome in the assembly to the phased SNP array data. Supplementary Table 1 provides information for which individuals reference SNP array data and accurate reference phasing information was available. For cultivars ‘Giambun’, ‘Kitchkova’, ‘Rouget’ and ‘Scilly Pearl’, SNP array data could not be accurately phased because only a few direct relatives were present. For ‘Ipador’ no reference SNP array data was available. For these reasons, results from the cultivars ‘Giambun’, ‘Kitchkova’, ‘Rouget’, ‘Scilly Pearl’ and ‘Ipador’ were excluded.

## Data Records

The genome assemblies have been deposited at GenBank under the accessions: ‘Braeburn’ haplotype 1, GCA_052939155.1^51^; ‘Braeburn’ haplotype 2, GCA_052939175.1^52^; ‘Coxs Orange Pippin’ haplotype 1, GCA_052938675.1^53^; ‘Coxs Orange Pippin’ haplotype 2, J GCA_052938685.1^54^; ‘Edward VII’ haplotype 1, GCA_052938595.1^55^; ‘Edward VII’ haplotype 2, J GCA_052938615.1^56^; ‘Enterprise’ haplotype 1, GCA_052939425.1^57^; ‘Enterprise’ haplotype 2, GCA_052939435.1^58^; ‘ Esopus Sptizenburg’ haplotype 1, GCA_052939395.1^59^; ‘ Esopus Sptizenburg’ haplotype 2, GCA_052939415.1^60^; ‘Fiessers Erstling’ haplotype 1, GCA_052938715.1^61^; ‘Fiessers Erstling’ haplotype 2, GCA_052938735.1^62^; ‘Giambun’ haplotype 1, GCA_052939355.1^63^; ‘Giambun’ haplotype 2, GCA_052939365.1^64^; ‘Giga ‘ haplotype 1, GCA_052939315.1^65^; ‘Giga ‘ haplotype 2, GCA_052939335.1^66^; ‘Granny smith’ haplotype 1, GCA_052939275.1^67^; ‘Granny smith’ haplotype 2, GCA_052939295.1^68^; ‘Jonathan’ haplotype 1, GCA_052939245.1^69^; ‘Jonathan’ haplotype 2, GCA_052939235.1^70^; ‘Kitchkova’ haplotype 1, GCA_052939185.1^71^; ‘Kitchkova’ haplotype 2, GCA_052939215.1^72^; ‘McIntosh’ haplotype 1, GCA_052939115.1^73^; ‘McIntosh’ haplotype 2, GCA_052939125.1^74^; ‘Milwa’ haplotype 1, GCA_052939075.1^75^; ‘Milwa’ haplotype 2, GCA_052939095.1^76^; ‘Prima’ haplotype 1, GCA_052939015.1^77^; ‘Prima’ haplotype 2, GCA_052938985.1^78^; ‘Priscilla’ haplotype 1, GCA_052938555.1^79^; ‘Priscilla’ haplotype 2, GCA_052938575.1^80^; ‘Rouget’ haplotype 1, GCA_052938935.1^81^; ‘Rouget’ haplotype 2, GCA_052938975.1^82^; ‘Scilly Pearl’ haplotype 1, GCA_052938915.1^83^; ‘Scilly Pearl’ haplotype 2, GCA_052938925.1^84^; ‘SQ59’ haplotype 1, GCA_052939035.1^85^; ‘SQ59’ haplotype 2, GCA_052939045.1^86^; ‘Tropical Beauty’ haplotype 1, GCA_052938535.1^87^; ‘Tropical Beauty’ haplotype 2, GCA_052938515.1^88^.

All raw data and assemblies have been deposited in NCBI under BioProject PRJNA1168485^89^ and Sequence Read Archive (SRA) SRP560690^90^. The BioProject accessions for each haplome are shown in Supplementary Table 1.

## Technical Validation

### Genome contiguity

**Fig. 4:**
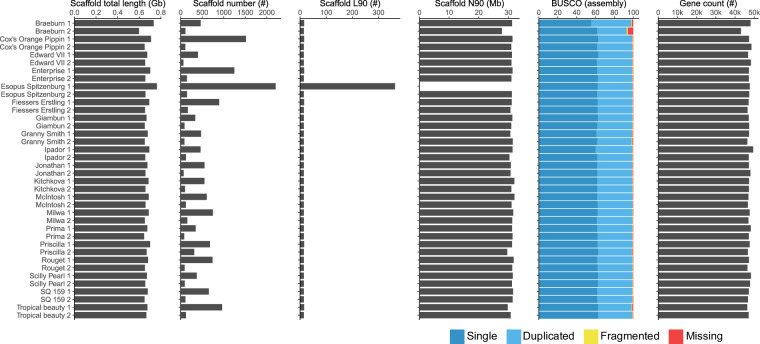
Barplot of the assembly statistics for each assembled haplome, showing total scaffold length, scaffold count, L90, BUSCO completeness, and predicted gene number.

The final haplome sizes, ranged between 592,406,660 to 676,548,961 bp, with a mean of 648,968,431 bp (Fig. 4, Supplementary Table 1). For all assemblies, 90% (N90) of the bases were allotted to 16–17 contigs, the only exception being haplotype 1 of ‘Esopus Spitzenbug’ (N90 = 365) (Fig. 4, Supplementary Table 1). These results indicate that most assemblies represent chromosome level assemblies.

The haplome assemblies, evaluated for completeness using BUSCO with the embryophyta_odb10 data, yielded complete BUSCO scores ranging between 93.1% to 99.3% with a mean of 98.8% (Fig. 4, Supplementary Table 1). While most of the genes were single copy ($\bar{x}$ = 62.0%) the remaining were mostly duplicated copies ($\bar{x}$ = 31.0%), which is expected in a species whose genome underwent a recent whole genome duplication^91^. However, the haplotype 2 assembly of cv. ‘Braeburn’ was relatively less complete, with fragmented copies and missing copies accounting for 1.3% and 5.6%, respectively. This was also confirmed in the comparison with the genetic map where only 81% of SNPs could be located (compared to approximately 94% for other assemblies). Given the high BUSCO scores we conclude that the assembled haplomes are of high completeness.

### Gene and protein content

The number of protein coding genes per haplome ranged between 43,278 and 49,666, with a mean of 47,445. This is greater than the doubled haploid GDDH13 v1.1^4^ genome (n = 42,140) and the triple haploid HFTH1 v1.0^5^ genome (n = 39,617) but more in line with that of the phased genomes of Gala v1.0 and *Malus sieversii* v1.0 and *Malus sylvestris* v1.0^10^ (n = 45,199-45,352).

### Orthogroups

Protein sequences were identified for 1,988,899 genes among 42 haplome assemblies (two consensus haploid assemblies and 20 cultivars with haplotype-resolved assemblies). In total 1,978,214 (99.46%) of the genes were assigned to 60,012 orthogroups and only 10,685 (0.54%) were singleton genes. There were 13,985 orthogroups where all assemblies were present, while there were 8,647 single-copy orthogroups. These data indicate 2,669 orthogroups are not found in HFTH1 v1.0, 1,010 orthogroups are not found in GDDH13 v1.1, and 578 orthogroups are not found in both HFTH1 v1.0 and GDDH13 v1.1, but are present in all others. An overall visualization of the results is illustrated in Supplementary Fig. 1. These data demonstrate the completeness (based on overlap) and novel diversity (based on abundance of single-copy orthogroups) of the genomes sequenced.

### Genome phasing quality check

**Fig. 5:**
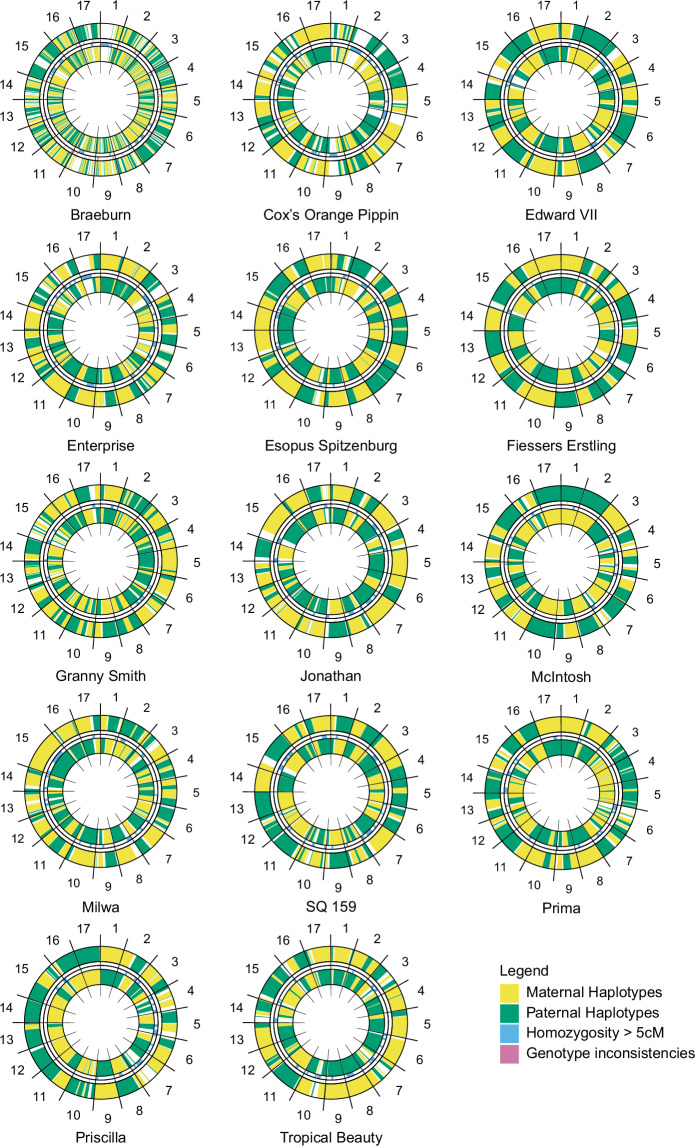
Visual representation of the phasing of 14 genome assemblies, with each circle representing a diploid genome assembly. Haplome 1 (inner) and Haplome 2 (outer) show parental origins (green/yellow), with intra-chromosomal color switches marking phase switches. Chromosomes are separated by black lines; homozygous segments >5 cM and genotype inconsistencies are shown as blue and pink bars, respectively.

To assess the quality of the phasing of the haplome assemblies, we compared the haplomes to SNP array data from individuals in an accession’s pedigree, for fourteen cultivars with complementary SNP array data. We found that, in general, phasing was mostly contiguous at a local level with phase switches occurring on average three to four times per chromosome (Fig. 5). For the cultivar ‘Braeburn’, the higher number of phase switches indicates a lower quality of phasing and is likely due to low sequence coverage. Therefore, this assembly could be improved in the future using more sequence data (Fig. 5). The phasing analysis indicates that the majority of haplomes provided here represent high quality phased genome assemblies. The challenges of phasing certain cultivars, for example due to lack of phased reference SNP data from direct relatives, indicates we have captured accessions that possess novel genetic diversity compared to popular breeding material that has already been sequenced. Overall, the genome assemblies presented here represent a resource for small scale local imputation. However, full chromosome scale imputation still requires further improvement in phasing.

## Supplementary information

Supplementary information

Supplementary Table 1

## Data Availability

All raw data and assemblies are available on NCBI under BioProject PRJNA1168485^89^.

## Code availability

The bioinformatic tools and software used for the analyses were executed according to their respective published manuals. The versions and parameters of each bioinformatic tool and software that was used are listed within the methods section.
