# Supplementary material for: Haplotype-resolved chromosome-level genome assemblies of nineteen apple (Malus domestica Borkh.) cultivars
Source: Sci Data. 2026 Jan 24;13:258. doi: 10.1038/s41597-026-06583-y (PMC12917252; doi:10.1038/s41597-026-06583-y)
Supplement: Supplementary file 1 — Supplementary information [file 41597_2026_6583_MOESM1_ESM.docx]

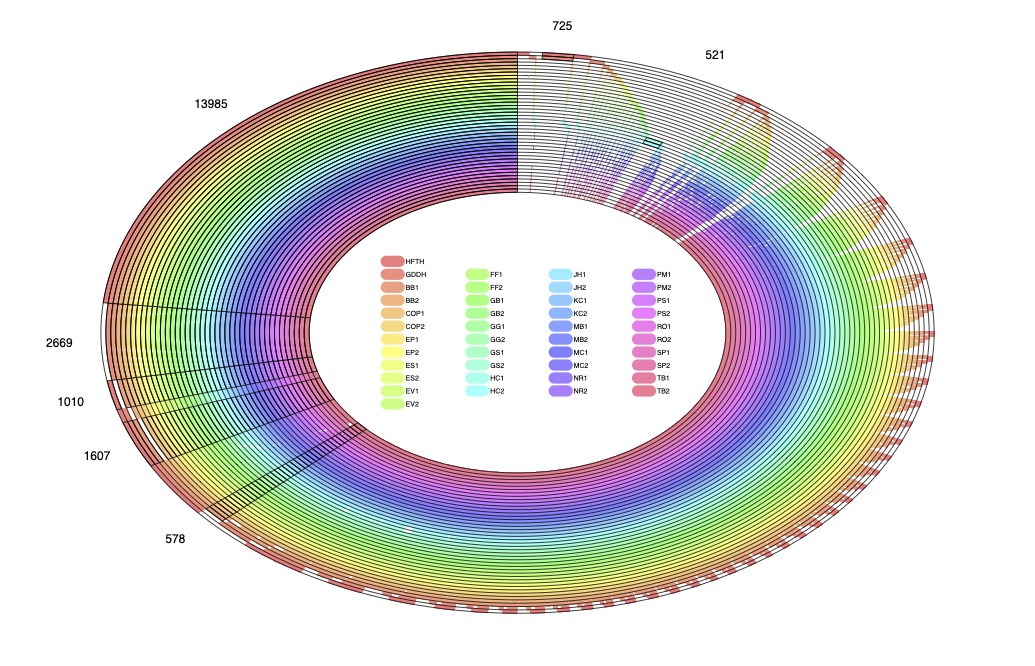


**Figure S1.** Ortholog analysis of protein coding genes predicted in the 38 haplotype resolved genomes described, the published haplomes for ‘HoneyCrisp’ (HC), and two reference genomes GDDH13 v1.1 and HFTH1 v1.0. The number outside the outer ring is the number of ortholog groups. White coloured sections indicate that there are no ortholog genes for that sample. The total number of orthogroups is 60,012. The haplomes are abbreviated with the following prefix , before haplome number: BB, 'Braeburn'; COP, 'Cox's Orange Pippin'; EP, 'Enterprise'; ES, 'Esopus Spitzenburg'; EV, 'Edward VII'; FF, 'Fiessers Erstling'; GB, 'Giambun'; GG, 'Ipador'; GS, 'Granny Smith'; JH1, 'Jonathan'; KC, 'Kitchovka'; MB, 'Milwa'; MC, 'McIntosh'; NR, 'SQ 159'; PM, 'Prima'; PS, 'Priscilla'; RO, 'Rouget'; SP, 'Scilly Pearl'; TB, 'Tropical Beauty’.
